# Organ-Specific Endothelial Dysfunction Following Total Body Irradiation Exposure

**DOI:** 10.3390/toxics10120747

**Published:** 2022-12-01

**Authors:** Guru Prasad Sharma, Heather A. Himburg

**Affiliations:** 1 Department of Radiation Oncology, Medical College of Wisconsin, Milwaukee, WI 53226, USA; 2 Cancer Center, Medical College of Wisconsin, Milwaukee, WI 53226, USA

**Keywords:** H-ARS, GI-ARS, delayed effects of acute radiation exposure (DEARE), total body irradiation, multi-organ injury, endothelial dysfunction, medical countermeasures

## Abstract

As the single cell lining of the heart and all blood vessels, the vascular endothelium serves a critical role in maintaining homeostasis via control of vascular tone, immune cell recruitment, and macromolecular transit. For victims of acute high-dose radiation exposure, damage to the vascular endothelium may exacerbate the pathogenesis of acute and delayed multi-organ radiation toxicities. While commonalities exist between radiation-induced endothelial dysfunction in radiosensitive organs, the vascular endothelium is known to be highly heterogeneous as it is required to serve tissue and organ specific roles. In keeping with its organ and tissue specific functionality, the molecular and cellular response of the endothelium to radiation injury varies by organ. Therefore, in the development of medical countermeasures for multi-organ injury, it is necessary to consider organ and tissue-specific endothelial responses to both injury and candidate mitigators. The purpose of this review is to summarize the pathogenesis of endothelial dysfunction following total or near total body irradiation exposure at the level of individual radiosensitive organs.

## 1. Introduction

Victims of whole or near whole body radiation exposure will experience multi-organ acute and late toxicities. This review focuses on the potential role of the vascular endothelium in the progression of organ specific sequelae following the type of high dose exposure that may be likely to occur in a catastrophic nuclear or radiological event [1]. While injury to the vascular endothelium is an important dose-limiting toxicity for radiotherapy (RT), these mechanisms have been reviewed elsewhere [2,3]. Additionally, whole or near whole body irradiation exposure introduces comorbidities that are likely to exacerbate tissue damage to each individual organ system [4]. For example, survivors of the atomic bomb are known to suffer from lifelong immune impairment that is likely to impact vascular function [5].

At the cellular level, ionizing radiation is known to induce immediate endothelial damage characterized by apoptotic cell death, the release of pro-inflammatory cytokines, expression of cellular adhesion molecules, and loss of endothelial barrier function [6,7]. Data from genetic endothelial gain and loss of function models suggest endothelial damage contributes to the severity of the acute radiation subsyndromes of the bone marrow [8,9] and gastrointestinal tract [10,11,12]. In addition to acute endothelial damage, ionizing radiation induces long-term functional changes in the endothelium which may contribute to the pathogenesis of a range of late morbidities including neurological, cardiopulmonary, gastrointestinal, and endocrine disorders. Here, we first review common pathological changes in the endothelium which may be relevant to both acute and late responding organs. We then focus on radiation injury at the tissue and organ level as the endothelium is known to be highly heterogeneous with tissue-specific differences in structure, phenotype, and function [13,14,15].

## 2. Radiation-Induced Endothelial Cell Injury

**Figure 1:**
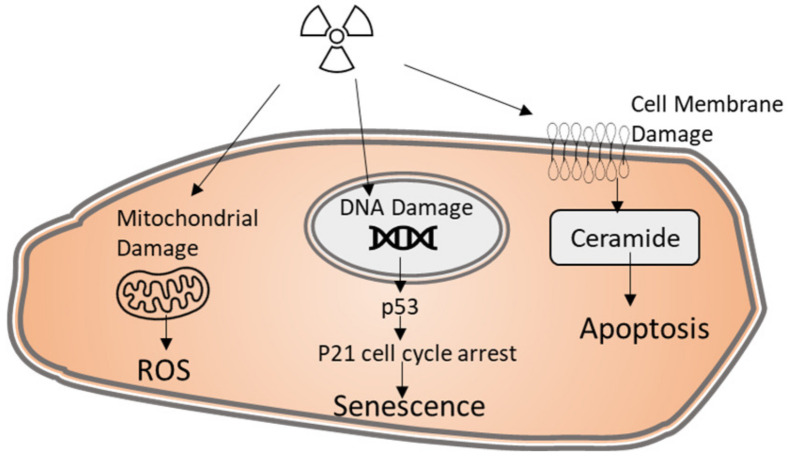
Schematic depiction of the main mechanisms of radiation-induced endothelial cell damage.

In general, ionizing radiation induces direct cellular injury via three mechanisms: induction of DNA damage, damage to the plasma membrane, and generation of reactive oxygen species (ROS) as illustrated in Figure 1. The formation of radiation-induced double stranded DNA breaks (DSBs) is known to increase linearly with radiation dose [16]. DNA damage is detected by the PI3K- related kinase (PIKK) family of kinases which includes ataxia-telangiectasia mutated (ATM) [17]. ATM then activates the DNA damage signaling pathway which includes activation of master transcription regulator p53. P53 regulates transcription of a number of downstream targets involved in cell growth arrest (p21), DNA repair, and the intrinsic apoptotic pathway [18]. P53-mediated induction of the intrinsic apoptotic pathway occurs in highly proliferative tissues such as the hematopoietic system, hair follicles, thymus, and intestinal epithelium, p53 [19].

In endothelial cells, radiation-induced endothelial p53 activation typically promotes p21-mediated cell cycle arrest and the development of a senescent cell phenotype [20]. Although viable, senescent cells are in permanent cell-cycle arrest and can be phenotypically characterized by increased beta-galactosidase activity (SA-B gal) [19]. Several models support radiation-induced senescence downstream of p53 activation in the endothelium. This was clearly evidenced in endothelial conditional p53 knockout models which showed loss of p53 led to an increase in endothelial radiosensitivity rather than resistance to apoptotic cell death [21]. This finding is also supported by in vitro data in pulmonary artery endothelial cells which showed accelerated cellular senescence is the primary cause of clonogenic cell death following in vitro radiation of pulmonary artery endothelial cells demonstrated by upregulation of p21, loss of SIRT1, and increased SA-B gal [22]. While the senescent endothelial cell is not capable of replication, the presence of senescent endothelial cells is thought to contribute to chronic inflammation and vascular dysfunction as senescent cells are known to exhibit inflammatory cytokines [23]. Consistent with this idea is the observation that radiation induces a concurrent increase in SA-B gal and monocyte adhesion [24].

In addition to DNA damage, ionizing radiation causes direct damage to the cell membrane causes direct damage to the cellular membrane by lipid peroxidation and fragmentation. Radiation induces hydrolysis of the membrane lipid sphingomyelin to ceramide by the membrane bound lipid enzyme acidic sphingomyelinase (ASMase) [10,25]. Ceramide accumulation in the membrane occurs within minutes of radiation injury [26] and has been shown to positively regulate both the extrinsic and intrinsic apoptotic pathways [27,28]. Early studies from the Kolesnick laboratory demonstrated sphingomyelin-ceramide mediated induced apoptosis is the primary mechanism for radiation-induced endothelial cell apoptosis. In these experiments, radiation induced the immediate conversion of sphingomyelin to ceramide which occurred independent of DNA damage [26]. Independence of the ceramide pathway from p53-mediated intrinsic apoptotic cell death was confirmed in genetic ASMase loss of function mouse models. In these studies, radiation-induced apoptosis of the endothelium was absent in the thymus, lung, and intestines of irradiated ASMase knockout mice [29,30,31].

Finally, radiation injury is known to induce the generation of ROS. Although ROS are generated within seconds following radiation injury [32], only a transient amount of ROS species are generated by the hydrolysis of water from ionizing radiation [33]. The majority of radiation-induced ROS species are thought to be generated by loss of mitochondrial integrity as cells lacking functional mitochondria do not produce ROS in response to radiation [34]. Radiation injury at lower doses (2 Gy in vitro exposure) has been shown to induce mitochondrial dysfunction without increasing intracellular ROS [35]. In this study, radiation reduced the number of endothelial mitochondria and altered mitochondrial structure [35]. Interestingly, pretreatment with the compound rosiglitazone which stimulates mitochondrial biogenesis and oxidative phosphorylation, protected mitochondria from radiation-induced injury and apoptotic death [35].

## 3. Radiation-Induced Endothelial Functional Alterations

**Figure 2:**
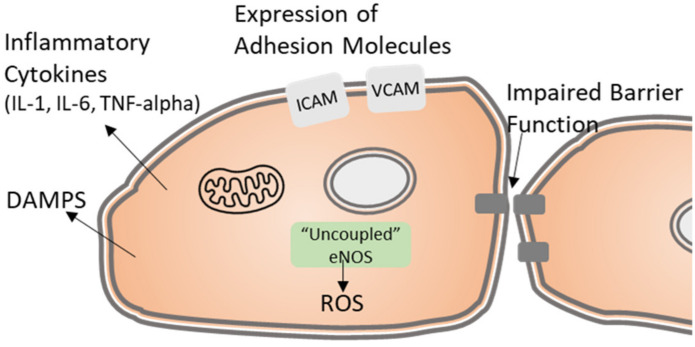
Schematic depiction of the mechanisms which contribute to radiation-induced endothelial cell dysfunction.

Endothelial cells which escape both radiation-induced senescence or apoptotic cell death, may have long-term functional impairment as illustrated in Figure 2 [23]. In their comprehensive review of radiation-induced endothelial injury, Baselet et al. describes the activation of the endothelium to a pro-inflammatory state in the absence of typical in vivo endogenous factors such as TNF-a and IL-6 which are known to induce endothelial activation. Such “sterile inflammation” is characterized by the release of multiple pro-inflammatory cytokines (IL-1, IL-6, TNF-alpha) and the cell-surface expression of immune cell-recruiting adhesion molecules (VCAM, ICAM, CD44). The induction of this pro-inflammatory state is mediated by a combination of radiation-induced mechanisms which include: DNA damage, release of damage-associated molecular patterns (DAMPs), and generation of reactive oxygen species (ROS). Each of these mechanisms is known to promote signaling cascades which lead to activation of the genotoxic stress-induced nuclear factor (NF)-κB pathway [36]. The NF-κB signaling cascade induces the expression of adhesion molecules ICAM, VCAM, and E-selectin and release of inflammatory cytokines [37,38].

The expression of cell adhesion molecules on the endothelium is a key step in the initiation and progression of inflammatory cardiovascular diseases as these adhesion molecules promote leukocyte adhesion and transendothelial migration of neutrophils [7]. Recent data from Soroush et al. demonstrate the serine/threonine kinase PKCδ regulates the radiation-induced expression of adhesion molecules P-selectin, ICAM-1, and VCAM-1 [39,40]. Radiation induces a dose-dependent phosphorylation and activation of PKCδ in endothelial cells exposed to physiological levels of fluid shear stress in a 3D microfluidic chamber. Importantly inhibition of PKCδ reduces transcription of adhesion molecules and reduces neutrophil adhesion [39,40]. Translationally, preclinical data suggest peptide inhibitors of PKCδ (PKCδ-TAT) improve survival in mouse H-ARS models [7].

Another important characteristic of functional impairment in the irradiated endothelium is a loss of endothelial barrier function. As will be discussed later, endothelial permeability varies in an organic-specific manner. In general, the endothelium serves as a selective barrier which regulates the transport of fluids, solutes, macromolecules, and cells from the blood to the tissue space. Barrier integrity is maintained by endothelial cell-specific junctional proteins of the adherens, tight, and gap junctions [41]. Several labs have documented radiation-induced impairment of endothelial barrier function in vitro and in vivo [39,42,43,44,45]. Impairment of barrier integrity following radiation injury has been associated with dysregulation of the key adherens junctional proteins platelet endothelial cell adhesion molecule (PECAM-1) and vascular endothelial cadherin (VE-Cadherin) [44,45]. Kouam et al. demonstrate a dose-dependent increase in endothelial monolayer permeability which occurs concurrently with degradation of the full-length form of VE-Cadherin and dislocalization of VE-Cadherin on the cell surface. Moreover, they demonstrate loss of VE-Cadherin is mediated by the metalloproteinase ADAM10 (a disintegrin and metalloproteinase) [44]. Additionally, radiation-induced regulation of endothelial integrity is regulated by serine/threonine kinase PKCδ [39].

Finally, dysfunction of the endothelium is generally associated with impaired endothelial nitric oxide synthase (eNOS) activity. Here, “uncoupled” eNOS favors production of the ROS super oxide (O_2_^-^) and peroxynitrite (ONOO−) instead of the vasodilator nitric oxide (NO). Superoxide dismutase (SOD) mimetics which act to scavenge ROS have shown promise in mitigation of lung and intestinal radiation injury [46,47,48]. More recently, work by Rabender et al. demonstrated treatment with sepiapterin, a cofactor for the production of nitric oxide (tetrahydrobiopterin (BH4)), mitigates against late radiation-induced lung and heart injury in mice [49].

## 4. Hematologic Acute Radiation Syndrome (H-ARS)

The hematopoietic system is acutely sensitive to radiation exposure. In human populations, whole body radiation doses greater than 2 Gy may lead to fatality 2–8 weeks following exposure due to acute bone marrow toxicity and the resulting loss of mature blood cells and platelets. Hematopoietic function including regeneration is tightly regulated by the bone marrow (BM) microenvironment which includes the endothelial cells (ECs) commonly divided into two vascular niches: the arteriolar and sinusoidal vessels. The arteriolar and sinusoidal endothelial cells defining these distinct vascular niches [50] are known to play highly specialized roles in the regulation of hematopoietic stem cells within the BM as reviewed by Ramalingam et al. [51].

Physically, the sinusoidal endothelium forms a network of permeable capillaries which serve to traffic nutrients and hematopoietic cells to and from the BM microenvironment [52]. Early transmission electron micrograph studies demonstrated sinusoidal ECs experienced membrane damage which results in loss of endothelial barrier function [53]. Subsequent studies have shown dose and time dependent changes including dilation of sinusoidal vessels, loss of sinusoidal vessels, increased permeability, and alterations in the molecular profile of the surviving EC fraction [54,55,56,57]. Relative to non-hematopoietic tissues including the retina, heart, skin, and small intestine, Chen et al. demonstrated the bone marrow sinusoidal endothelium is more sensitive to radiation injury [56]. Interestingly, the increased sensitivity of BM ECs to radiation may be due to reliance on the hematopoietic compartment for necessary EC growth factors such as VEGF as genetic depletion of hematopoietic cell compartment phenocopies radiation-induced vascular injury. Finally, Chen et al. also showed regeneration of the bone marrow sinusoidal endothelium is regulated by a rare and relative radiation-resistant population of sinusoidal ECs expressing the marker Apelin [56].

Bone marrow endothelial cells (BMECs) play a key role in restoring hematopoiesis following radiation injury in part by secretion of hematopoietic cytokines such as G-CSF, EGF, pleiotrophin, jagged-1, CCL5 and E-selection [13,58,59,60,61,62,63]. Indeed, genetic gain and loss of function models have demonstrated the necessity of the BM vascular niche for hematopoietic regeneration following radiation injury. Genetic protection of the endogenous endothelium from intrinsic apoptotic cell death is sufficient to rescue mice from lethal radiation injury [9]. Genetic or pharmacological inhibition of the sinusoidal endothelial-specific receptor VEGFR2 inhibits both structural recovery of sinusoidal vessels and reduces hematopoietic reconstitution [8]. Additionally, genetic deletion of endothelial secreted hematopoietic cytokines jagged-1 or pleiotrophin inhibits hematopoietic stem cell regeneration following radiation injury [58,64].

Collectively, these studies emphasize the fundamental significance of the endothelium in regulating hematopoietic regeneration following radiation injury. Accordingly, several studies have evaluated whether therapeutic delivery of endothelial cells can improve hematopoietic regeneration after injury. Multiple groups have demonstrated that infusion of endothelial cells or endothelial cell-derived extracellular vesicles (EVs) can accelerate hematopoietic recovery and rescues lethally irradiated mice from death due to hematopoietic syndrome [50,65,66,67,68,69]. Additionally, in an in vitro comparison, endothelial cell co-culture promoted better regeneration of hematopoietic stem cells than treatment with the established medical countermeasure (MCM) G-CSF alone and was efficacious even when treatment was delayed to 48 h [70].

In addition to endothelial cellular therapies, therapies targeting the endogenous bone marrow endothelium have also been evaluated as potential medical countermeasures for hematopoietic acute radiation syndrome. Notably, activated protein C (APC) is a factor which counters radiation-induced loss of endothelial thrombomodulin to reverse pro-thrombotic and pro-fibrogenic remodeling of the endothelium [71]. Among other mitigative roles discussed later, treatment with APC has been shown to reduce radiation-induced hematopoietic toxicity [72,73]. While the vascular endothelium exerts many protective and regenerative roles, in the context of injury it can release factors such as semaphorin 3A (sema3A) which promote endothelial cell apoptosis [74]. Targeted inhibition of this pro-apoptotic mechanism with an antibody specifically blocking sema3A binding to the endothelial receptor neuropilin 1 promotes concomitant endothelial & hematopoietic recovery [74].

## 5. Gastrointestinal Acute Radiation Syndrome (GI-ARS)

Persons exposed to whole body doses as low as 1.5 Gy may experience radiation injury to the gastrointestinal (GI) tract as evidenced by early symptoms of nausea, vomiting, and anorexia. Without treatment, doses above 5 Gy may result in GI death due to denudation of the intestinal mucosal barrier and subsequent fluid loss, hemorrhage, and/or sepsis [75,76]. The denudation of intestinal mucosal barrier results from an acute loss of the intestinal stem cell population which resides in the intestinal crypts and functions to replenish the epithelial layer [77]. Within the intestinal villi, intestinal endothelial cells form a dense vascular network within the gut epithelium. The intestinal vasculature plays a unique role as it serves as a selective permeability barrier (the gut vascular barrier or GVB) which allows the necessary absorption of nutrients while blocking the translocation of bacteria and large macromolecules [78].

In the setting of acute intestinal injury, it has been debated whether GI toxicity results from initiation of apoptotic death in the endothelium or the intestinal crypt [10,79]. While the relative contribution of the endothelium to the initiation of injury remains unclear, it is evident the intestinal endothelium plays a key role in the pathogenesis of acute GI injury as genetic protection of the intestinal endothelium against ceramide-mediated apoptotic cell death via the deletion of the acid sphingomyelinase gene [10] is protective against lethal GI toxicity. Subsequent studies have demonstrated this mechanism is critical to survival following lethal GI injury as pharmacologic neutralization of endothelial ceramide signaling promotes intestinal stem cell survival and effectively mitigates radiation-induced GI toxicity [11,12].

## 6. Late Responding Organs

Survivors of high-dose acute radiation exposure are at increased risk for developing a range of late multi-organ morbidities which are collectively referred to as the delayed effects of acute radiation exposure (DEARE). Sustained vascular dysfunction is a common trait which has been observed in multiple late radiation-sensitive tissues including the lung [80,81,82,83,84,85], kidneys [86,87], heart [88,89,90,91,92], brain [93], and gastrointestinal tract [71]. Late vascular dysfunction is characterized by structural changes in the vasculature including malformities [93], and vessel regression [81,82]. In a cohort of non-human primate (NHP) survivors of total body irradiation doses between 1.1–8.5 Gy at Wake Forest University, the incidence of vascular brain lesions was non-invasively observed with MR imaging for more than 10 years post-irradiation. While most of the cohort exhibited early brain lesions (12/16 NHPs), there was a notable increase in the number of new lesions during surveillance [93]. Histopathological analysis of deceased animals demonstrated MRI lesions were correlated with abnormalities of the cerebral vasculature and remodeling of the vascular wall [93]. Together, these data suggest that survivors of acute radiation exposure are at elevated risk of developing vascular injury for many years following exposure.

## 7. Radiation Enteropathy

In addition to life-threatening acute GI syndrome, survivors of acute radiation exposure may experience radiation enteropathy a late-radiation toxicity characterized by mucosal loss, fibrosis, and sustained vascular dysfunction [71]. The mechanism which initiates late injury is thought to be distinct from acute toxicity as early epithelial damage does not necessarily correlate with late intestinal fibrosis in animal models [94]. Recently, Lee et al. demonstrated that while endothelial deletion of p53 had no effect on the acute GI syndrome, loss of endothelial p53 resulted in an increased susceptibility to late injury characterized by increased vascular permeability, tissue hypoxia, and reduced intestinal length [95]. A key mechanism regulating vascular dysfunction in radiation enteropathy is the thrombomodulin-protein C system. In the intestinal microvasculature, endothelial thrombomodulin production is markedly reduced following radiation injury [96,97]. The radiation-induced deficiency of thrombomodulin both increases levels of thrombin and reduces activated protein C (APC) [71]. The sustained dysregulation of this signaling pathway leads to increased blood clotting, inflammation, and pro-fibrotic collagen production [71]. For this reason, pharmacologic studies to target these pathways have been evaluated and suggest either recombinant thrombomodulin or therapies which promote thrombomodulin expression can mitigate intestinal injury in a rat model [98,99]. Interestingly, although APC treatment has established mitigative effects for acute injury [73], long-term genetic upregulation of APC had little effect on the progression of late vascular injury [100].

## 8. Radiation-Induced Lung Injury

Radiation-induced lung injury (RILI) can occur in two phases: an acute inflammatory stage (pneumonitis) 8–16 weeks following exposure and a late fibrotic injury which can take months to years to evolve [101]. These are two distinct mechanisms of injury as fibrosis can occur independently of overt pneumonitis [102]. However, endothelial dysfunction is common to both injury phases and contributes to the pathogenesis of both pneumonitis and fibrosis. The lung microvascular ECs are highly specialized and exist in close proximity to the alveolar epithelium to facilitate gas exchange between the circulation and alveoli.

Radiation pneumonitis is primarily an inflammatory condition characterized by pulmonary edema, immune cell infiltration, and clinical symptoms of labored breathing/cough. Animal models have been instructive in characterizing the acute vascular changes during pneumonitis. At one month following 10 Gy thoracic irradiation, marked changes in the pulmonary vasculature have been observed including a loss of the pulmonary vessels and increased vascular resistance [82]. Concurrent with the loss in vasculature, increased vascular permeability is observed in the lung of irradiated rats at 6 weeks following high dose radiation exposure [43]. Marked improvement in vascular function and survival during pneumonitis have been observed in rats treated with the angiotensin converting enzyme (ACE) inhibitor lisinopril [43,103]. While this effect may be mediated in part by the well-established vasodilatory function of lisinopril, recent data also suggest ACE inhibition suppresses immune-mediated production of inflammatory cytokines [104].

Pathologic alterations in vascular permeability during pneumonitis are attributable in part to alteration of lung endothelial sphingolipids [105]. Similar to intestinal injury, radiation induces an increase in the pro-apoptotic sphingolipid ceramide relative to the barrier protective sphingolipid sphingosine 1-phosphate (S1P) in lung endothelial cells [106]. Consistent with this observation, genetic deletion of S1P results in exacerbation of radiation-induced loss of pulmonary endothelial barrier function and treatment with S1P analogs prior to radiation exposure protected against loss of barrier function [106]. Finally, it has been shown that treatment with soy isoflavones may mediate clearance of damaged, pro-inflammatory lung endothelial cells immune cells which express adhesion molecules and release the DAMPs including HMGB1 [107].

## 9. Heart Disease

Schultz-Hector and Trott provided a comprehensive review of the manifestation of cardiovascular diseases in both clinical cohorts exposed to heart irradiation and epidemiologic analysis of the Life Span Study of Japanese atomic bomb survivors [108]. This review highlighted the increased risk for development of a range of cardiovascular diseases including ischemic heart disease, arteriosclerosis, and cardiomyopathy following radiation exposure [108]. Atomic bomb survivors have been shown to have increased circulating levels of pro-inflammatory cytokines IL-6 and C-reactive protein (CRP) [109] which is suggestive of sustained and systemic endothelial dysfunction. In a long-term analysis of the Life Span Study cohort, doses above 0.5 Gy were associated with an elevated risk of both heart disease and stroke [110]. A higher incidence of cerebrovascular disease was also observed in the Mayak Production Association cohort comprised of 18,797 workers exposed at the Mayak nuclear facility [111].

## 10. Radiation Nephropathy

Survivors of acute radiation syndrome are at increased risk for developing late kidney injury or radiation nephropathy. In a cohort analysis of Japanese atomic bomb survivors, radiation dose was significantly associated with chronic kidney disease [112]. The glomerular endothelium has been identified as the primary site of radiation damage in the kidney [113]. The glomerular endothelium is another highly specialized endothelial cell type which functions to filter massive volumes of plasma through transcellular pores (fenestrae) [114]. Radiation injury induces structural changes to glomerular endothelium including swelling and separation from the basement membrane [113,115]. Functionally, this results in a decrease in glomerular filtration rate and a progressive decline in renal function. In a rat model, vascular regression in the kidney was associated with a loss of Notch ligand, jagged1. Treatment with the ACE inhibitor lisinopril has been observed to both improve renal function following radiation injury in rat models and normalize renal jagged1 expression [87].

## 11. Conclusions

Radiation-induced EC injury is a major mediator and regulator of wider tissue damage in the different vascular beds. The data reviewed here highlight the role of the highly heterogeneous endothelium in the progression of multi-organ injury. While the endothelium of radiosensitive organs is highly specialized, there may be common mechanisms which can be targeted to systemically promote normalization of the endothelium post radiation injury. Several aforementioned therapies (summarized in Table 1 below) include peptide inhibitors of PKCδ, exogenous endothelial cell products, ACE inhibitors, factors targeting the thrombomodulin-activated protein C pathway, anti-ceramide antibodies, S1P analogs, SOD mimetics, and sepiapterin.

## Figures and Tables

**Table 1:** Candidate Mitigators of Radiation-Induced Endothelial Cell Injury.

Mitigator	Mechanism of Action	Refs.
PKCδ-TAT	Blocks upregulation of adhesion molecules	[7,39,40]
SOD Mimetics	Scavengers of ROS	[46,47,48]
Sepiapterin	Restores eNOS “coupling” and reduces ROS	[49]
ECs/Vesicles	Restores hematopoiesis and rescues H-ARS lethality	[65,66,67,68,69,70]
Anti-Sema3A	Blocks SEMA3A binding to Neuropilin-1	[74]
APC	Corrects for thrombomodulin loss	[72,73,98,99]
ACE inhibitors	Reduces recruitment of inflammatory immune cells	[103,104]
Anti-ceramide	Blocks ceramide-mediated apoptotic death	[10,11,12]
S1P Analogs	Restores endothelial barrier function	[105,106]
Soy isoflavones	Promote clearance of damaged endothelium	[107]

## Data Availability

Not applicable.
